# Additive Effects of VDBP and 1,25(OH)2D3 on the Viability and Apoptosis of Rheumatoid Arthritis Synovial Fibroblasts

**DOI:** 10.3389/fendo.2020.583229

**Published:** 2021-01-28

**Authors:** Yeyong Zhang, Shufeng Li, Feng Zhuo, Hongxing Wang, Xiubin Geng, Bing Xu, Luxu Yin, Huaqiang Sun, Xinfeng Yan

**Affiliations:** ^1^ Department of Orthopedic Surgery, Shandong Qianfoshan Hospital, Cheeloo College of Medicine, Shandong University, Jinan, China; ^2^ Shandong Provincial Key Laboratory for Rheumatic Disease and Translational Medicine, Shandong Qianfoshan Hospital, Cheeloo College of Medicine, Shandong University, Jinan, China; ^3^ Department of Orthopedic Surgery, Tai’an Central Hospital, Tai’an, China; ^4^ Cheeloo College of Medicine, Shandong University, Jinan, China; ^5^ Department of Orthopedic Surgery, Jinan Municipal Third Hospital, Jinan, China

**Keywords:** vitamin D-binding protein, 1,25(OH)2D3, proliferation, apoptosis, rheumatoid arthritis synovial fibroblast

## Abstract

**Aim:**

This study is to investigate the additive effect of Vitamin D-binding protein (VDBP) and 1,25(OH)2D3 on the viability and apoptosis of synovial cells from patients with rheumatoid arthritis (RA).

**Methods:**

Synovial tissues and synovial fluid of patients with RA and osteoarthritis (OA) were collected. The expression of VDBP was analyzed with immunohistochemistry and ELISA. CCK-8 assay was applied to detect cell viability. Flow cytometry was used to analyze cell cycle and apoptosis.

**Results:**

Immunohistochemical results showed that the expression of VDBP in the synovium of RA patients was significantly lower than that of OA (P<0.05). Similarly, ELISA results presented a lower expression of VDBP in the synovial fluid of RA patients. The results of CCK-8 assay showed that both 1,25(OH)2D3 and VDBP significantly inhibited the viability of rheumatoid arthritis synovial fibroblasts (RASF) (P<0.05). The treatment with 1,25(OH)2D3+VDBP led to more significantly inhibited viability of RASF, compared with 1,25(OH)2D3 alone (P<0.05). The results of flow cytometry showed that 1,25(OH)2D3 and VDBP both promoted the apoptosis of RASF (P<0.05) and 1,25(OH)2D3+VDBP led to a higher proportion of RASF apoptosis, compared with 1,25(OH)2D3 alone (P<0.05). However, 1,25(OH)2D3 and VDBP had no significant effect on the cell cycle of RASF. Additionally, 1,25(OH)2D3 promoted the expression of VDBP in RASF, but not concentration-dependently.

**Conclusion:**

VDBP is reduced in the synovial tissue and synovial fluid of RA patients and can inhibit viability of RASF and promote the apoptosis of RASF. The 1,25(OH)2D3 can upregulate the expression of VDBP in RASF. Additionally, VDBP can enhance the effects of 1,25(OH)2D3 on viability and apoptosis of RASF.

## Introduction

Rheumatoid arthritis (RA) is a chronic autoimmune disease characterized by synovial hyperplasia, inflammatory cell infiltration, pannus formation, destruction of articular cartilage and bone matrix, and symmetry or destructive development, which can eventually lead to joint deformity and loss of function (1). It has long disease course, high teratogenic rate and high disability rate. Because of its unclear etiology, there is no satisfactory clinical treatment plan for RA. Therefore, studying the pathological mechanism of RA has vital clinical significance.

The main active form of vitamin D is 1,25-dihydroxyvitamin D3 (1,25-(OH)2D3), which can regulate calcium and phosphorus metabolism in the human body (2). In clinic, the immune regulation ability of RA patients is significantly related to the adjuvant treatment of 1,25(OH)2D3. 1,25(OH)2D3 supplement can relieve pain for RA patients (3) and the incidence of RA can be reduced after an increase of 1,25(OH)2D3 intake (4). The level of 1,25(OH)2D3 in serum is significantly higher in stable or mild RA patients than moderate and severe RA patients, revealing that the1,25(OH)2D3 level is closely associated to disease activity of RA (5).

Vitamin D-binding protein (VDBP) is the transporter of 1,25(OH)2D3, which can transport 1,25(OH)2D3 between blood and cell membranes (6). It is mainly synthesized in the liver and widely distributed in various body fluids (such as plasma, cerebrospinal fluid, semen, saliva, and breast milk) (7, 8). It is also expressed in immune cells such as T lymphocytes, monocytes, macrophages, and dendritic cells (9), as well as in articular chondrocytes and synoviocytes at the site of RA injury (10). Our previous study showed that VDBP was significantly reduced in the synovial tissue of RA patients (11). However, the additive role of VDBP and 1,25(OH)2D3 in rheumatoid arthritis synovial fibroblasts (RASF) is less studied.

Herein, we aim to investigate the additive effects of VDBP and 1,25(OH)2D3 on the viability and apoptosis of synovial cells from RA patients with rheumatoid arthritis. Our findings may provide novel strategies and directions for the treatment of RA.

## Materials and Methods

### Subjects and Tissue Collection

Patients with RA (n=3, age 50 ± 10 years, female) and osteoarthritis (OA) (n=3, age 65 ± 10 years, female) who received knee joint replacement in 2018 at Qianfoshan Hospital of Shandong Province were enrolled. Human synovial tissue samples were collected during knee joint replacement surgery. The patients with RA had disease durations of 5–8 years and were classified as having erosive RA (Larsen class IV-V) (11). The patients with RA had stopped the medications of glucocorticoid and disease-modifying anti-rheumatic drugs two months before surgery. The patients with RA and OA were only treated with non-steroidal anti-inflammatory drugs, which helped to reduce the pain and swelling of the joints and decrease stiffness.

Synovial fluid samples were collected from patients with RA (n=16; 12 females and four males; average age 49 years) and OA (n=12; nine females and three males; average age 67 years). The synovial fluid samples were immediately stored at −80°C until use.

All RA and OA patients enrolled were diagnosed according to the classification criteria (2010 and 1995 edition, respectively) revised by the American College of Rheumatology (ACR). OA was used as a control for RA, as described previously (12, 13). Written informed consents were obtained from all subjects and the study was approved by the ethics review board of Qianfoshan Hospital, Shandong Province.

### Isolation and *In Vitro* Culture of RASF and OASF

The RASF and OASF cells were isolated as previously reported (12, 13). Briefly, the freshly collected synovial tissues were rinsed three times with PBS, cut into 1 mm3 pieces and then subjected to digestion with 4% type II collagenase (1 mg/ml) in 3 ml DMEM (Dulbecco’s Modified Eagle Medium; GE Healthcare-HyClone). After digestion for 4–6 h, a cell suspension was obtained, which were then filtered with a cell strainer (70–100 μm pore size) and centrifuged at 1,000 rpm for 5 min. The cell pellet was re-suspended and digested with trypsin for 0.5h. After centrifugation at 1,000 rpm for 5 min, cell pellet was re-suspended with DMEM containing 10% fetal bovine serum (Gibco). The cells were seeded in a culture flask, and cultured in a 5% CO2 incubator at 37°C. When the cells grew to 80% confluence, they were digested with 0.25% trypsin and subcultured to three to five generations, which were used for subsequent experiments. The RASF and OASF cells were confirmed with inverted phase contrast microscope.

### CCK-8 Assay

The 3^rd^-5^rd^ generations of human RASF cells were inoculated in a 96-well plate at 2,000 cells/well, and cultured in a 37°C incubator for 2 to 4h. Different concentrations of 1,25(OH)2D3 (0, 1, 5, 10, 50, and 100 nmol/L) (Abcam) or VDBP (0, 1, 5, 10, 50, and 100 ng/ml) (VDBP variant GC*1F; Cat# TP720729, OriGene Technologies) were added for incubation. After 72h, 10 μl CCK-8 (Dojindo) was added and incubated for 2h. Then, the absorbance at 450 nm (OD450) was measured with a microplate reader (Thermo Multiskan).

### Flow Cytometry for Apoptosis Analysis

The 3^rd^–5^rd^ generations of human RASF cells were seeded into six-well plates at 2–5×10^5^ cells/well. When the cell confluence reached about 75%, the experimental groups were respectively added with 10 nmol/L 1,25(OH)2D3, 5 ng/ml VDBP, and 10 nmol/L 1,25(OH)2D3 + 5 ng/ml VDBP. The control group was treated with an equal amount of PBS. After incubation for 72h, cells were collected and subjected to centrifugation. The 100 μl 1× binding buffer was added to re-suspend cells and then 5 μl Annexin V and 10 μl PI were added in sequence. After incubation at room temperature in the dark for 15min, 400 μl Binding buffer was added. Finally, flow cytometry (Beckman Coulter) was used to detect cell apoptosis.

### Flow Cytometry for Cell Cycle Analysis

Similarly, 3^rd^–5^rd^ generations of human RASF cells were seeded into six-well plates at 2–5×10^5^ cells/well. When the cell confluence was about 75%, 10 nmol/L 1,25(OH)2D3, 5 ng/ml VDBP, and 10 nmol/L 1,25 (OH) 2D3 + 5 ng/ml VDBP were added. The control group was treated with an equal amount of PBS. After 72h, cells were collected and fixed with ice-cold 75% ethanol at 4°C overnight. After washing with 1 ml ice-cold PBS twice, cells were added with 500 μl RNaseA (20 μg/ml) for incubation at 37°C for 30min. After washing again, cells were treated with 500 μl PI (propidium iodide) (50 μg/ml) at room temperature in the dark for 30min. Cell cycle was detected with a flow cytometer (Beckman Coulter).

### Western Blot

The 3^rd^-5^rd^ generations of human RASF cells were seeded into six-well plates at 2–5×10^5^ cells/well for incubation until the cell confluence reached about 75%. The cells were treated with 1,25(OH)2D3 (0,1,10,100 nmol/L), and the control group was incubated with the same amount of PBS. After 72h, cells were collected. Protein samples were extracted after cell lysis. The proteins were separated by electrophoresis, and transferred to the PVDF membrane. After blocking with 5% skimmed milk for 1.5–2 h at room temperature, the primary antibody against VDBP (Abcam) and anti-GAPDH was added and incubated overnight at 4°C. After that, the membranes were treated with diluted secondary antibody (Invitrogen) and incubated at 37°C for 1h. After washing, the membrane was developed by enhanced chemiluminescence. GAPDH was used as an internal reference. The gray value was analyzed with Image J.

### Immunohistochemical Analysis

Synovial tissues were embedded and sectioned. After antigen retrieval by heat, the sections were blocked with 5% BSA at room temperature for 20min. After washing, primary antibody against VDBP (Abcam) was added and incubated at 37°C for 1h. Then, the sections were treated with secondary antibody (Invitrogen) at 37°C for 15–30 min. The color was developed with DAB. After that, the sections were counterstained with hematoxylin for 2min. At last, the sections were mounted and observed under an optical microscope (Nikon).

### Enzyme-Linked Immunosorbent Assay (ELISA)

ELISA detection kit from MultiScience was used. Briefly, 100 μl synovial fluid sample and standard sample were separately added to the plate and incubated at 4°C overnight. After washing for 5 times, 10 μl VDBP antibody was added and incubated at 4°C for 1h. After washing again, 100 μl HRP labeled antibody was added and incubated at room temperature for 45min. After that, the plate was added with 100 μl TMB chromogenic solution and incubated at room temperature for 10–30min. The absorbance was detected at 450 nm wavelength. A standard curve was drawn to calculate the VDBP concentration.

### Statistical Analysis

The experiments were performed separately on samples from each patient. Each experiment was repeated three times. Data were expressed as Mean **±** SD. The SPSS19.0 statistical software was used for the statistical analysis. The paired t-test was performed for data comparison. P **<**0.05 was considered as statistically significant.

## Results

### Immunohistochemical Analysis of VDBP in RA Synovium and OA Synovium

**Figure 1:**
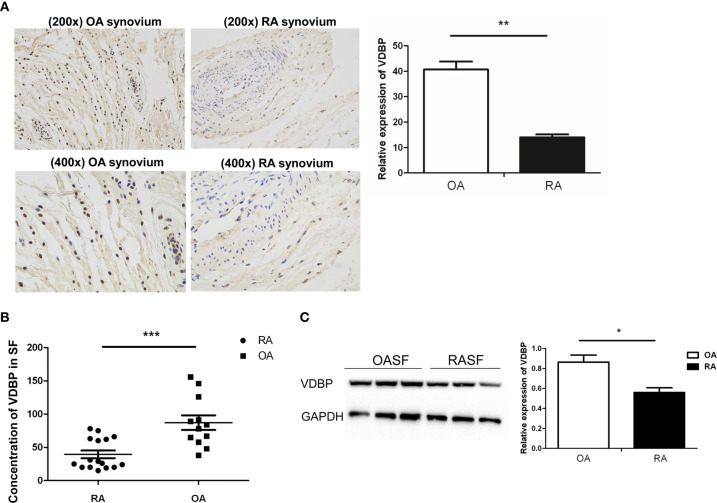
Analysis of Vitamin D-binding protein (VDBP) expression. **(A)** Expression of VDBP in synovium of osteoarthritis (OA) and rheumatoid arthritis (RA) patients. Immunohistochemistry was performed to detect VDBP expression. Representative immunohistochemistry results (at 200 x and 400 x magnification) and relative level of VDBP were shown. **(B)** The level of VDBP in synovial fluid of RA and OA patients. Enzyme-linked immunosorbent assay (ELISA) was conducted to detect VDBP in synovial fluid. **(C)** VDBP protein level in OASF and rheumatoid arthritis synovial fibroblasts (RASF). Western blot was carried out to detect protein expression in three RA patients and three OA patients. The representative Western blot results and the relative expression of VDBP from western blot were shown. Each lane represents the sample from an individual patient. Several western blots were used for quantification. *P < 0.05, **P < 0.01, ***P < 0.001.

Immunohistochemical staining was performed to analyze VDBP expression in synovium from RA patients (n=3) and OA patients (n=3). As shown in **Figure 1A**, VDBP staining in OA synovium was stronger than in RA synovium. Statistical analysis showed that the expression level of VDBP in OA synovium was higher (P<0.05).

### Level of VDBP in RA Joint Fluid and OA Joint Fluid

ELISA was applied to detect VDBP level in the synovial fluid of RA and OA patients. The results showed that the level of VDBP in the synovium of RA patients was significantly lower than that of OA patients (**Figure 1B**).

### Western Blot Analysis of VDBP Expression in RASF and OASF

We isolated and cultured the synovial fibroblasts (RASF and OASF) from three RA patients and three OA patients. Western blot results demonstrated a significantly lower expression level of VDBP protein in RASF than that of OASF (**Figure 1C**).

### Effect of VDBP on Viability of RASF

**Figure 2:**
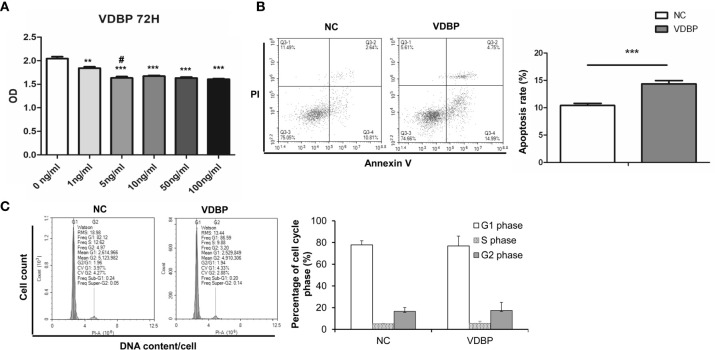
Effect of Vitamin D-binding protein (VDBP) on viability, apoptosis and cell cycle of RASF. **(A)** Effect of VDBP on viability of rheumatoid arthritis synovial fibroblasts (RASF). Human recombinant VDBP (0, 1, 5, 10, 50, 100 ng/ml) was added to RASF for 72 h. Cell viability was assessed with CCK-8 assay. Compared with 0 ng/ml, **P < 0.01, ***P < 0.001. Compared with 1 ng/ml, ^#^P < 0.05. **(B)** Apoptosis analysis of RASF cells with or without 5 ng/ml VDBP treatment. RASF cells were treated without (NC) or with 5 ng/ml VDBP for 72 h. Flow cytometry was used to detect apoptosis. Representative and quantitative flow cytometry results were shown. ***P < 0.001. **(C)** Cell cycle analysis of RASF cells treated with or without 5 ng/ml VDBP. RASF cells were treated without (NC) or with 5 ng/ml VDBP for 72 h. Flow cytometry was used to detect cell cycle. Representative and quantitative flow cytometry results were shown.

Different concentrations of human recombinant VDBP (0, 1, 5, 10, 50, 100 ng/ml) were used to treat RASF for 72 h. Then, CCK-8 assay was conducted to detect cell viability. The results showed that VDBP at 1, 5, 10, 50, and 100 ng/ml significantly inhibited the viability of RASF, compared with the control group (0 ng/ml VDBP) (P<0.05, **Figure 2A**). Compared with cells treated with 1 ng/ml, those treated with 5 ng/ml had significantly lower viability. However, at other concentrations (10, 50, and 100 ng/ml), the inhibitory effect on RASF viability was not significantly enhanced along with the increase of VDBP concentration (P>0.05).

### Effect of VDBP on the Apoptosis and Cell Cycle of RASF

After treating RASF with VDBP (5 ng/ml) for 72 h, cell apoptosis was detected with flow cytometry. Compared with the control group, 5 ng/ml VDBP promoted RASF apoptosis (P<0.05, **Figure 2B**), while had no significant effect on RASF cell cycle (P>0.05, **Figure 2C**).

### Effect of 1,25(OH)2D3 on Viability of RASF

**Figure 3:**
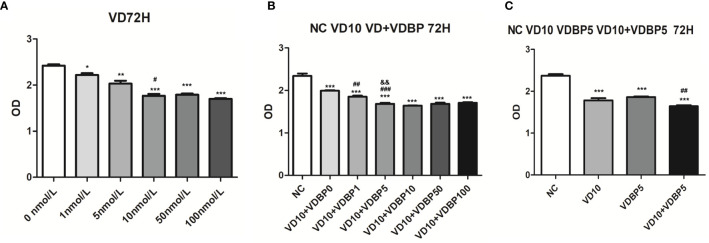
Effect of 1,25(OH)2D3, Vitamin D-binding protein (VDBP), and 1,25(OH)2D3+VDBP on RASF. **(A)** The effect of 1,25(OH)2D3 on the viability of rheumatoid arthritis synovial fibroblasts (RASF) cells. RASF cells were treated with different concentrations of 1,25(OH)2D3 (0,1,5,10,50,100 nmol/L) for 72 h. Cell viability was assessed with CCK-8 assay. Compared with 0 nmol/L, *P < 0.05, **P < 0.01, ***P < 0.001. Compared with 5 nmol/L, ^#^P < 0.05. **(B)** The enhanced inhibition of 1,25(OH)2D3 with VDBP on the viability of RASF. RASF cells were treated with 1,25(OH)2D3+VDBP as indicated in the figure. Compared with NC, ***P<0.001. Compared with VD10+VDBP0, ^##^P < 0.01, ^###^P < 0.001. Compared with VD10+VDBP1, &&P<0.01. **(C)** The enhanced inhibition of 1,25(OH)2D3 with VDBP on the viability of RASF. RASF cells were treated with 1,25(OH)2D3, VDBP, and 1,25(OH)2D3+VDBP as indicated in the figure. Compared with NC, ***P < 0.001. Compared with VD10, ^##^P < 0.01.

Different concentrations of 1,25(OH)2D3 (0, 1, 5, 10, 50, and 100 nmol/L) were used to treat RASF for 72 h. CCK-8 assay results showed that compared with the control group (0 nmol/L 1,25(OH)2D3), 1,25(OH)2D3 at the concentration of 1, 5, 10, 50, and 100 nmol/L significantly inhibited the viability of RASF (P<0.05, **Figure 3A**). The cell viability at 10 nmol/L was significantly lower than that at 5 nmol/L (P<0.05). However, at 50 and 100 nmol/L, the inhibitory effect on cell viability was not further significantly changed than that at 10 nmol/L (P>0.05).

### Enhanced Inhibition of 1,25(OH)2D3 with VDBP on the Viability of RASF Cells

To analyze the combined effect of 1,25(OH)2D3 and VDBP on cell viability, RASF were treated with 10 nmol/L 1,25(OH)2D3 and different concentrations of human recombinant VDBP (0, 1, 5, 10, 50, 100 ng/ml) for 72 h. All the 1,25(OH)2D3+VDBP groups had statistically significantly inhibitory effects on the viability of RASF, compared to the control group and 1,25(OH)2D3 alone group (10 nmol/L) (**Figure 3B**). Among them, the groups treated with 10 nmol/L 1,25(OH)2D3 + 5 ng/ml VDBP and 10 nmol/L 1,25(OH)2D3 + 10 ng/ml VDBP had similar levels in inhibiting the viability of RASF (**Figure 3B**).

RASF cells were respectively treated with 10 nmol/L 1,25(OH)2D3, 5 ng/ml VDBP, and 10 nmol/L 1,25(OH)2D3+5 ng/ml VDBP for 72 h. CCK-8 assay showed that both 1,25(OH)2D3 and VDBP had significantly inhibitory effects on the viability of RASF, compared to the control group. This effect was enhanced by the combined use of 1,25(OH)2D3+VDBP (**Figure 3C**). This result indicates that VDBP can enhance the role of 1,25(OH)2D3 in the inhibitory effects on the viability of RASF.

### VDBP Promotes the Effect of 1,25(OH)2D3 on Apoptosis and Cell Cycle of RASF Cells

**Figure 4:**
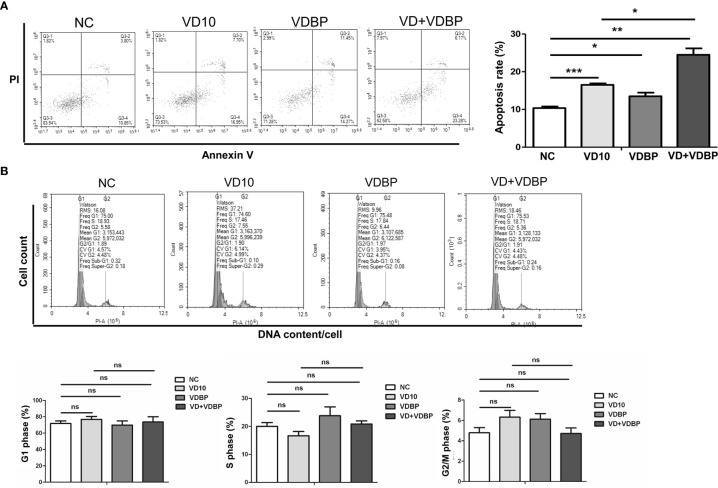
Flow cytometry analysis of apoptosis and cell cycle. **(A)** Apoptosis analysis of rheumatoid arthritis synovial fibroblasts (RASF) after treatment with 1,25(OH)2D3, VDBP and 1,25(OH)2D3+VDBP. RASF cell treatment was indicated in the figure. Flow cytometry was used to detect apoptosis. Representative and quantitative flow cytometry results were shown. *P<0.05, **P<0.01, ***P<0.001. **(B)** Cell cycle analysis of RASF after treatment with 1,25(OH)2D3, VDBP and 1,25(OH)2D3+VDBP. RASF cell treatment was indicated in the figure. Flow cytometry was used to detect cell cycle. Representative and quantitative flow cytometry results were shown. ns, not significant.

RASF were respectively treated with 10 nmol/L 1,25(OH)2D3, 5 ng/ml VDBP, and 10 nmol/L 1,25(OH)2D3+5 ng/ml VDBP for 72 h. Flow cytometry was performed to detect apoptosis and cell cycle. As shown **Figure 4A**, both 1,25(OH)2D3 and VDBP had pro-apoptotic effect on RASF cells and this effect was enhanced by the combined use of 1,25(OH)2D3+VDBP (P<0.05). Compared with the 1,25(OH)2D3 group, the pro-apoptotic effect of 1,25(OH)2D3+VDBP group was stronger, indicating that VDBP can enhance the role of 1,25(OH)2D3 in promoting RASF cell apoptosis (P<0.05).

However, cell cycle results revealed that 1,25(OH)2D3, VDBP and 1,25(OH)2D3+VDBP had no significant effect on the numbers of RASF in G0/1, S or G2 phase(P>0.05), which demonstrates VDBP and 1,25(OH)2D3 have no significant effect on cell cycle of RASF (P>0.05) (**Figure 4B**).

### Effect of 1,25(OH)2D3 on the Expression of VDBP

**Figure 5:**
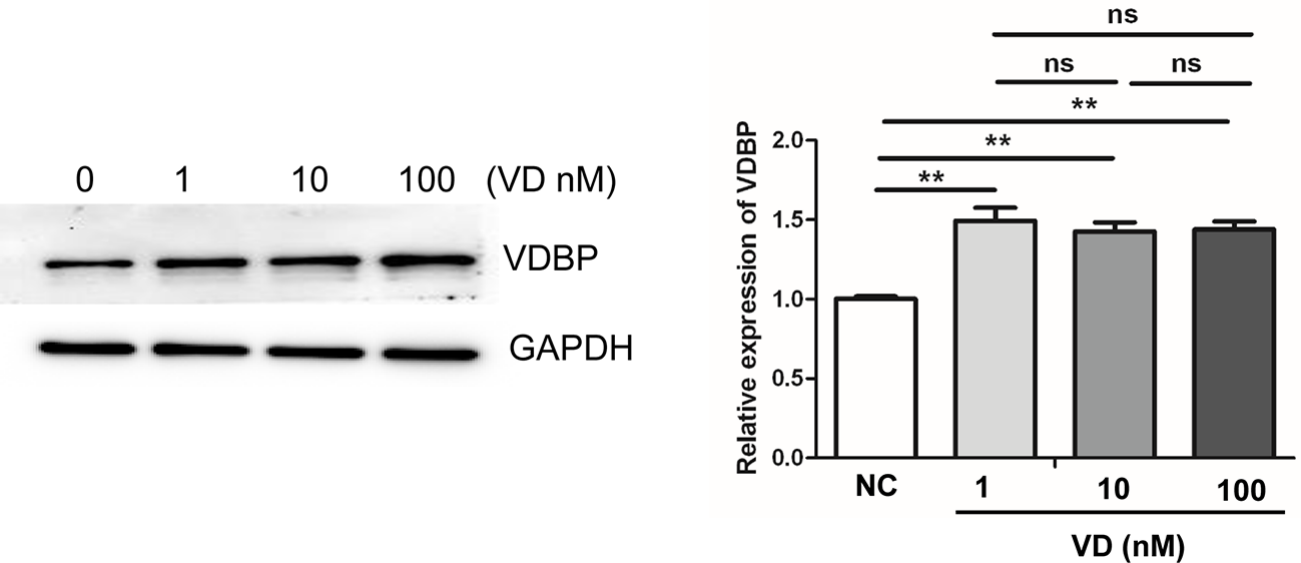
Effect of 1,25(OH)2D3 on the expression of Vitamin D-binding protein (VDBP). Rheumatoid arthritis synovial fibroblasts (RASF) was treated with different concentrations of 1,25(OH)2D3. Western blot was used to detect VDBP protein expression. The representative Western blot results and the relative expression of VDBP from western blot were shown. **P < 0.01. ns, not significant.

Western blot analysis was performed after treating RASF with different concentrations of 1,25(OH)2D3 (0, 1, 10, 100 nmol/L) for 72 h. The findings indicated that the addition of 1,25(OH)2D3 to RASF could promote the expression of VDBP (P<0.05); however, with the increase of 1,25(OH)2D3 concentration, the expression of VDBP did not increase accordingly (P>0.05), suggesting there is no dose-dependent effect (**Figure 5**).

## Discussion

RA is a chronic autoimmune disease characterized by severe inflammation and joint destruction and it affects approximately 1% of the world’s population (14). Hyperplasia of RASF is the main cause of synovial hyperplasia and plays an important role in the development of RA. Similar to cancer cells, RASF also have the features of abnormal proliferation and apoptosis. Therefore, how to effectively inhibit excessive proliferation of synovial cells and induce their apoptosis is a potential therapeutic strategy for RA therapy (15).

VDBP, also known as GC globulin, is synthesized in the liver and has a variety of physiological functions including carrying vitamin D metabolites in the circulation, binding to actin (16), transporting fatty acids (17), and acting as a macrophage activation factor (18). Even though VDBP has important roles in many diseases, its function in RA still remains inconclusive. Our previous study showed that RA patients had significantly reduced VDBP in the synovial tissue (11). This study further validated our previous findings by showing that the expression of VDBP in the synovial tissue and synovial fluid of RA patients was less than that of OA patients. RASF and OASF are the main cells in the synovium of RA and OA patients. The results also showed that the expression of VDBP in RASF was reduced compared to that in OASF. In order to explore the role of VDBP in RASF, RASF was treated with VDBP. CCK8 assay and flow cytometry analysis were performed to study the effects of VDBP on the viability, cell cycle and apoptosis of RASF, respectively. The results revealed that VDBP could inhibit viability of RASF and promote the apoptosis of RASF without affecting cell cycle, suggesting that VDBP may play a therapeutic role in RA by promoting the apoptosis of RASF.

sVitamin D, as a vital vitamin or essential dietary component, has a wide range of biological effects, such as regulating the calcium and phosphorus metabolism to promote a healthy mineralization, mediating the growth and remodeling of bone, as well as regulating the antibacterial activity and cell differentiation (19). In addition, vitamin D has an inhibitory effect on human cells and adapts immune response, which is directly related with the development of RA (20–22). Some studies have found that low vitamin D levels can be accompanied by increased RA activity and promote procession of RA (23–28). It is also reported that supplement of vitamin D and their analogues can relieve RA in most patients (29). Furthermore, 1,25 (OH) 2D3 is found to take an important part in immune regulation and disease improvement in arthritis mouse models (30). However, not all studies hold the same point of view (31–34), and whether vitamin D could affect RA’s incidence and activity is still debatable (35). In this study, we treated RASF with 1,25(OH)2D3 and found that 1,25(OH)2D3 inhibited the viability of RASF, which is consistent with the results that the biologically active forms of 1,25(OH)2D3 and its calcitriol analogs can strongly inhibit the growth of synovial fibroblasts *in vitro* (12). This verifies that 1,25(OH)2D3 can inhibit the viability of RASF and may play a positive role in the treatment of RA.

It is reported that the expression of VDBP in RA patients was lower than that of OA patients (11). However, the role of VDBP in the treatment of RA has not been reported. In order to evaluate the therapeutic effect of VDBP in the treatment of RA with 1,25(OH)2D3, we used 1,25(OH)2D3 and VDBP together on RASF. We found that 1,25(OH)2D3 could inhibit the viability of RASF and promote the apoptosis. Compared with 1,25(OH)2D3 alone, the combination of 1,25(OH)2D3 and VDBP further promoted cell apoptosis and strengthened cell viability inhibition. These results indicate that VDBP can not only transport vitamin D and its analogs in the human body (6), but also play an additive role with 1,25(OH)2D3 in RASF.

In this study, we found that 1,25(OH)2D3 promoted the expression of VDBP in RASF, but with the increase of 1,25(OH)2D3 concentration, the expression of VDBP did not increase accordingly. This indicates that 1,25(OH)2D3 can stimulate the expression of VDBP in RASF. However, there was not a concentration-dependent linear relationship between 1,25(OH)2D3 concentration and VDBP expression. On the other hand, this suggests that the effect of VD+VDBP on the viability and apoptosis of RASFs may be mainly mediated by the externally added VDBP but not the VDBP induced by vitamin D. Our result was in part inconsistent with the previous result that oral administration of large doses of vitamin D did not increase VDBP level in circulation (36). This may because different experimental systems were used. Vitamin D can be produced by liver and kidney hydroxylation in the human body after irradiated by ultraviolet rays (37). Since a certain amount of 1,25(OH)2D3 is already existing in the body, an exogenous intake of vitamin D will not further increase the expression of VDBP in the body. It has also been reported that the total concentration of 25OHD correlated positively with both VDBP and albumin; the percent free 25OHD correlated negatively with VDBP and albumin; and the free 25OHD levels did not correlate with either VDBP or albumin (38). Another study has shown that serum levels of directly measured free 25(OH)D positively correlated with total 25(OH)D (39). Therefore, the effect of 1,25(OH)2D3 on VDBP is still inconclusive and needs further investigation.

Additionally, it has been reported that 1,25(OH)2D3 may exert pro-apoptotic and anti-proliferative effects by regulating NF-κB signaling pathway (40, 41). However, whether the pro-apoptotic and anti-proliferative effects of vitamin D and VDBP on RASF cells are mediated by NF-κB signaling pathway is unclear in this study. Further studies are warranted to clarify this.

In conclusion, this study found that the expression of VDBP in the synovium and synovial fluid of RA patients was reduced. VDBP enhanced the effects of 1,25(OH)2D3 on viability and apoptosis of RASF. Additionally, 1,25(OH)2D3 increased the expression of VDBP in RASF cells, but not 1,25(OH)2D3 concentration-dependently. Therefore, our findings provide a new theoretical basis for the application of VDBP in enhancing 1,25(OH)2D3’s treatment effect on RA, inhibiting the synovial hyperplasia of RA patients, reducing RA bone destruction, and improving prognosis. Follow-up animal experiments and large-scale clinical observations are needed to provide more objective and comprehensive results to support the preliminary conclusions in this study.

## Data Availability Statement

The raw data supporting the conclusions of this article will be made available by the authors, without undue reservation.

## Ethics Statement

The studies involving human participants were reviewed and approved by the ethics review board of Qianfoshan Hospital, Shandong Province. The patients/participants provided their written informed consent to participate in this study.

## Author Contributions

YZ, SL: Data collection, data interpretation, manuscript preparation. FZ: Statistical analysis. HW, XG, BX, LY: Data collection. HS: Literature search. XY: Study design, funds collection. All authors contributed to the article and approved the submitted version.

## Funding

This work was supported by the Natural Science Foundation of Shandong Province (No. ZR2019MH130).

## Conflict of Interest

The authors declare that the research was conducted in the absence of any commercial or financial relationships that could be construed as a potential conflict of interest.
